# Prediction of daily and cumulative cases for COVID-19 infection based on reproductive number (R_0_) in Karnataka: a data-driven analytics

**DOI:** 10.1038/s41598-021-89573-x

**Published:** 2021-05-12

**Authors:** Kuralayanapalya Puttahonnappa Suresh, Sharanagouda S. Patil, Bharath Prasad Cholanayakanahalli Thyagaraju, Srikantha Gowda Ramkrishnappa, Divakar Hemadri, S. Chandrashekara

**Affiliations:** 1 Department of Spatial Epidemiology, ICAR-National Institute of Veterinary Epidemiology and Disease Informatics, Bengaluru, Karnataka India; 2 Department of Virology, ICAR-National Institute of Veterinary Epidemiology and Disease Informatics, Bengaluru, Karnataka India; 3 ChanRe Rheumatology and Immunology Centre and Research, Bengaluru, Karnataka India

**Keywords:** Computational biology and bioinformatics, Microbiology, Health care

## Abstract

To estimate the reproductive number (R_0_) of the coronavirus in the present scenario and to predict the incidence of daily and probable cumulative cases, by 20 August, 2020 for Karnataka state in India. The model used serial interval with a gamma distribution and applied ‘early R’ to estimate the R_0_ and ‘projections’ package in R program. This was performed to mimic the probable cumulative epidemic trajectories and predict future daily incidence by fitting the data to existing daily incidence and the estimated R_0_ by a model based on the assumption that daily incidence follows Poisson distribution. The maximum-likelihood (ML) value of R_0_ was 2.242 for COVID-19 outbreak, as on June 2020. The median with 95% CI of R_0_ values was 2.242 (1.50–3.00) estimated by bootstrap resampling method. The expected number of new cases for the next 60 days would progressively increase, and the estimated cumulative cases would reach 27,238 (26,008–28,467) at the end of 60th day in the future. But, if R_0_ value was doubled the estimated total number of cumulative cases would increase up to 432,411 (400,929–463,893) and if, R_0_ increase by 50%, the cases would increase up to 86,386 (80,910–91,861). The probable outbreak size and future daily cumulative incidence are largely dependent on the change in R_0_ values. Hence, it is vital to expedite the hospital provisions, medical facility enhancement work, and number of random tests for COVID-19 at a very rapid pace to prepare the state for exponential growth in next 2 months.

## Introduction

Coronavirus disease (COVID-19), a novel virus originated from Wuhan a city in the Hubei Province of China at the end of 2019, has progressed rapidly to become a global epidemic. In February 2020, the World Health Organization (WHO) designated the disease as COVID-19 and declared it as a global pandemic, as the disease has spread to nearly all the continents and the cases are rising at an exponential rate^1^.

The present study is aimed at predicting the spreading efficiency of the COVID-19 in Karnataka, which is one among the 28 states of India. The state has a total population of 6.41 crore, which is ~ 4.7% of the overall population of India. The first case of the COVID-19 in India was reported on January 30, 2020 in a couple who had a travel history to Dubai^2^. In Karnataka, the first case was detected on March 09, 2020^3^. On March 12, 2020 the World Health Organization (WHO) announced COVID-19 as a global pandemic to emphasize on the rapid spread of the disease to multiple countries and continents^4^.

As on June 23, 2020 the state’s case fatality rate was 1.01%, which was significantly lower than the global average and other Indian states with moderate number of cases^5^. Karnataka can be considered as moderately affected state with 9399 confirmed cases, 5730 recovered and 142 deaths^6^. More than two-thirds of the cases in the state have emerged from the migrant travelers from other states, mainly Maharashtra, Tamil Nadu, Delhi, Gujarat etc.

Restricted space and high population density of the country are the key factors influencing the transmissibility of COVID-19. The forecasting of the probable number of cases is essential to create awareness and arrange effective disease control measures^7^. There is a major threat associated with increase in disease spread, as most of the population belongs to below poverty line and the country does not have huge resources for medical interventions proportional to the population. The options to manage the disease are acquiring herd immunity and implementing lockdown or restricting the population movement^8^. In the present study, we have calculated the reproductive number (R_0_) of COVID-19 at an early stage of viral disease outbreak, to predict the daily incidence and cumulative cases for the next sixty days (till August 20, 2020).

## Methodology

### Focus

A confirmed case of COVID-19 infection is defined as those with a positive result for viral infection and history of acute respiratory illness for the collected specimens. A suspected case is defined as a patient with symptoms of COVID-19 infection, but not confirmed by viral nucleic acid testing. An actual estimate of the serial interval was considered by estimating the time from onset of illness in a primary case (infector) to illness onset in a secondary case (infected) in a transmission chain^9^. Serial interval can only be estimated by linking dates of onset for infector-infected data pairs, and these links are difficult to be established. R_0_ is defined as the actual expected number of secondary cases that one primary case will generate in a susceptible population^10^.

### Data source

All the data were derived from cloud sourced database published in the official website of Ministry of Health and Family Welfare of India^11^. The data for model development were updated from April 14, 2020 to June 21, 2020. However, the initial data were not considered, as the serial interval did not reflect the average behavior for effectively modelling the epidemic curve and number of effective cases was very low due to imposing strict lockdown in the state.

### Model development and statistical analysis

To estimate the contiguous transmissibility of COVID-19 in the state, the study employed the ‘early R’ statistical package to evaluate the R_0_ in the early stage of the disease outbreak^12^. *R* refers to the average number of secondary infected cases by one primary infected person during the infectious period. If *R* > 1, the number of cases increases; if *R* < 1, the number of infected cases reduces, and the disease will die out. When *R* = 1, it suggests that infectious disease has become an endemic within the community.

*R* is calculated by the probability of the spread of infection upon contact with an infected person, based on the number of contacts and the duration within the infected person that can help to spread the infection. Here, *β* refers to the probability of infection transmitted (transmission rate) multiplied by the contact levels and 1/α is the duration of infection transmitted. The mathematical model used to estimate *R* for COVID-19 in this study is represented below:

$$ R = \frac \beta \alpha $$

Serial interval (SI) distribution data was calculated to estimate R_0_, and there was inadequate information about total number of cases to estimate serial interval. The value of serial interval (mean and standard deviation) was fixed with a gamma distribution, as earlier described^13^. The ‘get_R’ function was used to estimate the distribution of R_0_ with maximum-likelihood (ML). A bootstrap strategy with 500 times resampling was adopted to get larger set of likely R_0_ values. These R_0_ values were subsequently presented in a histogram format, calculated the cumulative cases and interquartile range for these values. R package of ‘projections’ was used to estimate the probable epidemic trajectories simulation, prediction of future daily incidence and cumulative cases^14^. The simulation and prediction were generated by fitting the data to an existing daily incidence, a serial interval distribution, and the estimated R_0_ by a model based on the assumption that daily incidence followed Poisson distribution determined by daily infectiousness, which is denoted as

$$ \lambda t = \mathop \sum \limits_{s = 1}^{t - 1} ysw (t - s) $$

*w *(*t – s*) is the vector of probability mass function (PMF) of serial interval distribution and *y*_*s*_ is the real-time incidence at *s* time^14,15^. The study also computed the prediction of daily incidence and cumulative cases for the next 60 days using a bootstrap resampling method (500 times). All the statistical analysis and disease forecast model was developed by using R version 3.6.3.

## Results

**Figure 1:**
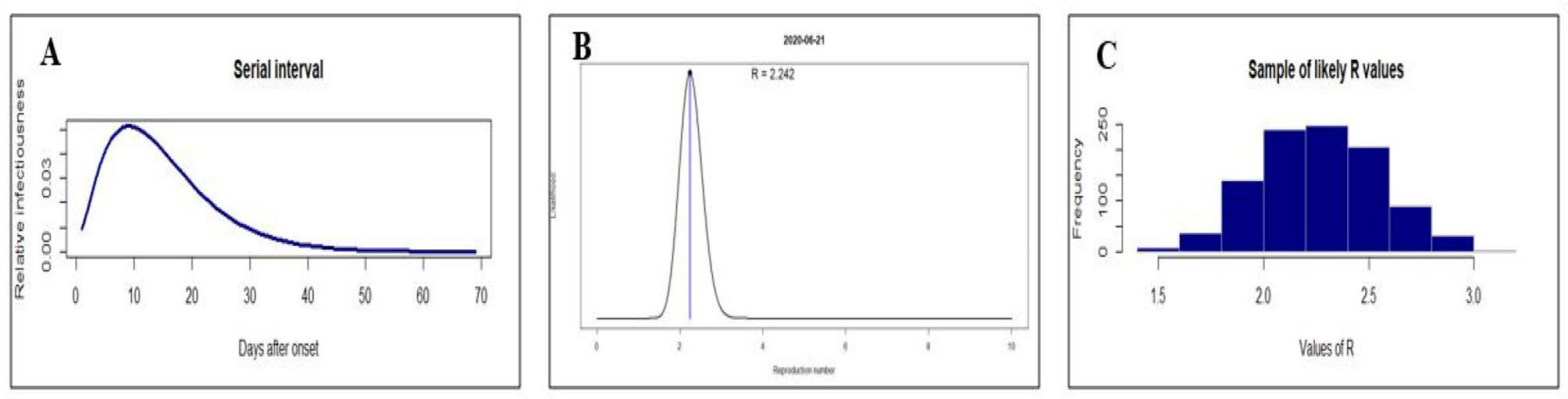
The distribution of serial interval (**A**), the present likely value of reproductive number (R_0_) estimated by the Maximum-Likelihood (ML) method (**B**), a sample of 500 likely R_0_ values using a bootstrap resampling method (**C**) for COVID-19 in Karnataka.

The COVID-19 serial interval distribution is shown in Fig. 1A. Using the serial interval distribution, as described above, the maximum likelihood estimate (MLE) value of R_0_ was found as 2.242 for COVID-19 outbreak at the present stage in Karnataka (Fig. 1B). Bootstrap strategy was adopted to obtain 500 likely R_0_ values. The distribution of these R_0_ values was presented as histogram plot (Fig. 1C). The estimated median with 95% confidence interval (CI) of R_0_ values was 2.242 (1.50–3.00), as on June 21, 2020.

**Figure 2:**
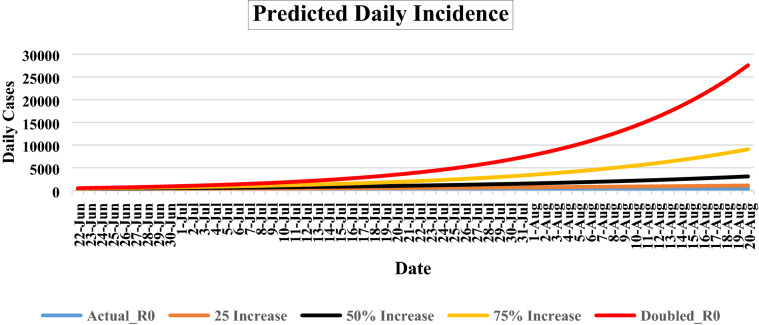
The probable epidemic trajectories of predicted daily cases for COVID-19 in Karnataka, India, June 22, 2020 and August 20, 2020. Red, yellow, black, orange line shows the probable daily cases, if the R_0_ value is doubled, 75%, 50% and 25% respectively. Blue line shows the probable number of daily cases, if R_0_ value was unchanged, for the next 60 days.

The probable number of daily new cases for the next 60 days was calculated based on existing data (Fig. 2). The daily number, at seven days’ interval (between June 22, 2020 and August 20, 2020) with 95% confidence interval (CI) of probable new cases at actual R_0_ (2.242) will be 255 (200–310), 250 (195–305), 253 (202–303), 261 (207–315), 277 (221–333), 293 (244–361), 312 (254–370), 339 (280–398), 356 (294–418), 378 (303–453), 414 (332–496), respectively. If the R_0_ value is presumed to increase by 25% (2.802), 50% (3.363) and 75% (3.923); the corresponding predicted daily new cases will be 1100 (970–1230), 3085 (2807–3362), and 9080 (8452–9708). If the R_0_ (4.484) gets doubled (100%) than actual R_0_, the daily new cases will drastically reach up to 27,831 (25,729–29,932) at the 60th day (August 20, 2020).

**Figure 3:**
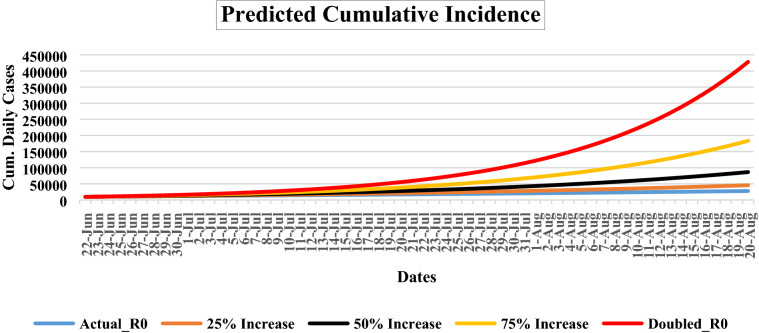
The probable epidemic trajectories of predicted cumulative cases for COVID-19 in Karnataka, India, June 22, 2020 and August 20, 2020. Red, yellow, black, orange line shows the probable daily cases, if the R_0_ value was doubled, 75%, 50% and 25% respectively. Blue line shows the probable number of cumulative cases, if R_0_ value was unchanged, for the next 60 days.

The range of cumulative number of cases for the next 60 days is shown in Fig. 3. The probable cumulative number of cases at seven days’ interval (between June 22, 2020 and August 20, 2020) with 95% CI at actual R_0_ (2.242) will be 9405 (9350–9460), 10,832 (10,646–11,018), 12,318 (12,032–12,604), 13,860 (13,496–14,223), 15,498 (15,009–15,987), 17,187 (16,604–17,769), 19,034 (18,358–19,710), 20,998 (20,192–21,803), 23,067 (22,092–24,041), 25,261 (24,130–26,391), 27,238 (26,008–28,467), respectively. If R_0_ (2.802) value is presumed to increase by 25% (2.802), 50% (3.363) and 75% (3.923); the corresponding predicted cumulative incidences will be 45,635 (43,011–48,258), 86,386 (80,910–91,861), and 184,167 (172,572–195,762). If R_0_ (4.484) gets doubled (100%) than actual R_0,_ the probable cumulative number of cases will reach up to 432,411 (400,929–463,893) at the end of prediction date (August 20, 2020).

## Discussion

Using the current data and the proposed pandemic model, the current study provides an estimation of the R_0_ for COVID-19 during the present stage of disease spread in Karnataka. The estimated distribution of R_0_ is about 2.242 (95% CI 1.50–3.00), which is similar to a set of previously published estimates, ranging from 2.286 (95% CI 1.4–3.9) to 3.58 (95% CI 2.89–4.39). It is equivalent to the reported estimate from a recent study with a larger sample size, which suggested the R_0_ of 3.77 (95% CI 3.51–4.05)^4,16,17^. The wide ranges and the difference in R_0_ values reported by different studies indicates that exact estimation of R_0_ is quite challenging, because it is difficult to calculate the exact total number of infected cases during an epidemic. The R_0_ value is usually affected by a set of factors like analysis, environmental circumstances, modeling procedures and statistical caliber^18^.

In the present study, the accuracy of estimated R_0_ is mainly dependent on the identification of all the infected cases in Karnataka. According to the report from Ministry of Health and Family Welfare of India, all the suspected cases and cases who had close contact with confirmed cases contracted viral infection after testing. Therefore, the percentage of unidentified cases is thought to be very low. In contrast, previous studies were mainly focused on the estimation of R_0_ in Wuhan, China^4,16^. Moreover, in contrast to such studies, the disease that can be transmitted from animals to individuals for SARS-Co V was absent among the extensively larger population of India^4,9^. Therefore, the R_0_ estimated in the current study only depicts the human-to-human transmissibility of COVID-19 and not from animals to human transmissibility. In Karnataka, the community level transmission has already begun and cases could be rising in the next 2 months^19^. The current results have concluded that the R_0_ is low due to enforcement of strict quarantine and lockdown measures in Karnataka. The current estimations also denote the silent spread of the virus at an exponential rate. Most of the patients, who are testing positive, are asymptomatic. Such cases within incubation may also cause continuous spread of the novel virus and this may also partially explain why low R_0_ was noted at current stage^19^.

The current study has also estimated the daily incidence, cumulative cases, and the probable size of the outbreak for the next sixty days. According to the analysis, the daily incidence and the magnitude of outbreak are largely dependent on the value of R_0_. If the R_0_ (2.242) value is presumed to remain unaffected, the probable cumulative number of infected cases may reach 27,238 (26,008–28,467) at the 60th day, suggesting more population would be infected in future. Karnataka has adopted strict measures to control the spread of infection by enforcing strict lockdown and quarantining the suspected cases. As a result, the transmissibility is expected to reduce in the future days. But, due to relaxation in lockdown, improper social distancing, and population mixing would lead to natural process of disease transmission, infected cases might increase in future due to change in R_0_ value. If the R_0_ (2.802) value increased by 50%, the infected cases will increase up to 86,386 (80,910–91,861), and if, R_0_ (3.923) increased by 75%, the infected cases will rise up to 184,167 (172,572–195,762). The scenarios such as opening of schools and colleges, migration of travelers from highly infected states, unhygienic condition at markets and crowded places, increase in religious conglomeration, and opening of overseas travelling may contribute to increase in number of active cases at rapid pace. This may lead to doubling of R_0_ (4.484) than actual R_0_ (2.242) values and the infected probable cumulative cases will reach up to 432,411 (400,929–463,893) at the end of 60th day (20 August, 2020). The present data-driven analytics is mainly aimed at predicting the probable epidemic size. It also highlights the importance of controlling the transmissibility among population, to prepare the state by arranging all medical facilities and resources to manage the estimated exponential increase.

R_0_ is not an intrinsic characteristic value of SARS-CoV pathogen, but it describes the transmissibility of that pathogen within the specific population and settings. Hence, R_0_ mainly depends on socio-demographic variable and the biology of infectious agent. Serial interval indicates that COVID-19 infection leads to rapid cycles of transmission from one generation of cases to the next. The difference between these distributions suggests that using serial interval estimates from SARS data will result in overestimation of the COVID-19 basic reproduction number and correct ascertainment on dates of illness onset is critical to calculate the serial interval^20,21^.

The current trend shows that there will be a geometric progression in the upcoming days due to relaxation of lockdown and negligent attitude of citizens regarding the infection spread, such as not using face mask, improper social distancing, mixing of more population, going to unhygienic market places, and not following strict advices given by the public health officials to consult doctors. Although the patient recovery rate is rising, the current trends indicate unprecedented increase in number of daily new cases and death rate due to COVID-19 infection. Even though stringent control measures are being implemented by the Government, there is increased chances of following the predicted geometric progression pattern, as the new cases identified in state are mainly in healthcare workers, police, BMTC, KSRTC and railway staffs, auto-rickshaw drivers, footpath vendors, delivery boys and salesmen. Such individuals may serve as super spreaders and it is essential to identify such hospital-based outbreaks and community-based clusters through continuous testing. Hospital provisions (in both public and private sectors) and medical facility enhancement work and number of random tests for COVID-19 infection should be continued at a very rapid pace to prepare the state for managing the predicted exponential growth. Through such current interventions and preparations, the Government of Karnataka is looking forward to flatten the pandemic curve.

## Conclusion

At the present stage of infection in Karnataka, the estimated R_0_ with 95% CI for COVID-19 is about 2.242 (1.50–3.00). The future daily incidence, probable cumulative cases and outbreak size are mainly dependent on value of R_0_. Due to the relaxation in lockdown, negligent attitude of people in maintaining social distancing, not taking precautionary measures and population mixing may accentuate the natural process of disease transmission. The number of active cases may double in the forthcoming days due to change in R_0_ value. The present findings highlight the importance of reducing transmissibility in controlling the probable outbreak size as well as to enhance the hospital provisions and medical facility (number of random tests) at very rapid pace to prepare the state for managing the worst situation for the months of September and October.
